# *Aster pekinensis* Extract Mitigates High-Fat-Diet-Induced Obesity and Metabolic Dysfunction in Mice

**DOI:** 10.3390/ani16020163

**Published:** 2026-01-06

**Authors:** Hyeon Jeong Moon, Seon-Jin Lee, Geon Woo Kim, Yeong-Bin Baek, Sang-Ik Park

**Affiliations:** 1 Department of Veterinary Pathology, College of Veterinary Medicine and BK21 FOUR Program, Chonnam National University, Gwangju 61186, Republic of Korea; dals404@naver.com (H.J.M.); sunjin002042@gmail.com (S.-J.L.); 2 Department of Integrative Food, Bioscience and Biotechnology, Graduate School of Chonnam National University, Gwangju 61186, Republic of Korea; 3 TWELL Inc., Yeongcheon 38882, Republic of Korea; ceo@twell.kr; 4 Department of Veterinary Pathology, College of Veterinary Medicine, Chonnam National University, Gwangju 61186, Republic of Korea

**Keywords:** *Aster pekinensis*, high-fat diet, obesity, non-alcoholic fatty liver disease, insulin resistance, triterpenoid saponins, nutraceutical, companion animals

## Abstract

Obesity and metabolic disorders are an increasing concern in companion animals, creating demand for herb-derived nutraceuticals and functional feeds. This study evaluated whether a water extract of *Aster pekinensis* (AP) ameliorates high-fat-diet (HFD)-induced obesity and metabolic dysfunction in mice. The phytochemical profile of AP was characterized by mass spectrometry, revealing oleanane-type triterpenoid saponins and dicaffeoylquinic acids. Male C57BL/6 mice were fed an HFD and orally given AP (10–200 mg/kg/day) for 12 weeks, with normal diet and untreated HFD groups as controls. AP at 50–200 mg/kg/day reduced body-weight gain, adipose tissue mass and food efficiency without lowering food intake, and improved fasting glucose and atherogenic lipid indices. AP also enhanced glucose tolerance and insulin sensitivity, attenuated hepatic steatosis, hepatocellular ballooning, lobular inflammation and non-alcoholic fatty liver disease (NAFLD) Activity Score, and decreased serum liver enzyme activities. These effects were accompanied by modulation of hepatic genes involved in lipogenesis and inflammation. Together, these findings indicate that AP extract mitigates diet-induced obesity and NAFLD-like liver injury and supports further development as a herb-derived nutraceutical or functional feed ingredient for managing obesity-related metabolic disorders in companion animals.

## Simple Summary

Obesity in dogs and cats is now one of the most common medical problems seen by veterinarians and is associated with chronic inflammation, fatty liver disease, and insulin resistance. Nutraceuticals and herbal extract-based functional foods are being explored as long-term adjuncts to help manage obesity-related metabolic problems in pets. Aster pekinensis (AP) is an East Asian herbal plant rich in triterpenoid saponins and caffeoylquinic acids with potential metabolic benefits. In this study, we used a high-fat diet mouse model that is widely employed to mimic obesity and fatty liver disease in companion animals to evaluate the phytochemical profile and metabolic effects of a water extract of AP. Mice were fed a high-fat diet for 12 weeks and given AP once daily at different doses. AP attenuated body-weight gain, reduced abdominal fat, improved blood sugar control and serum lipid profiles, and decreased liver fat and inflammatory markers in the liver and adipose tissue. These results suggest that AP could be further developed as a herb-derived component of functional diets or nutraceutical supplements for overweight dogs and cats.

## 1. Introduction

Obesity in companion animals has become increasingly prevalent in recent decades, mirroring trends observed in human populations. Recent practice-based surveys suggest that approximately 40–60% of dogs and cats in industrialized countries are overweight or obese, and the prevalence continues to rise in both primary care and referral hospital settings [1]. In dogs and cats, excess body fat not only reduces quality of life but is closely associated with a range of metabolic disorders, including insulin resistance, type 2 diabetes mellitus (T2DM), dyslipidemia, and non-alcoholic fatty liver disease (NAFLD) [2,3]. These conditions represent interconnected facets of metabolic syndrome, contributing to chronic inflammation, oxidative stress, and progressive organ damage. Beyond metabolic derangements, obesity is linked to reproductive disorders (e.g., dystocia) and impaired immune competence, which increase susceptibility to infectious diseases such as urinary tract infections [4]. Collectively, these observations underscore that obesity is not a cosmetic issue but a chronic, multifactorial disease that adversely affects longevity, welfare, and, in breeding animals, overall productivity. A key mechanistic feature is low-grade, chronic inflammation arising from immunometabolic crosstalk among adipocytes, hepatocytes, and tissue-resident immune cells [5]. In contrast to many livestock species, obesity-associated type 2 diabetes mellitus and NAFLD are particularly prevalent and clinically important in dogs and cats, making companion animal obesity an attractive target for evidence-based nutritional, functional-feed and herb-derived interventions.

Despite the serious clinical and welfare implications of obesity, current management strategies for companion animals face significant practical limitations. The primary interventions—caloric restriction, prescription weight-loss diets, and increased physical activity—are often difficult for owners to implement and sustain over the long term [6]. Normalization of excess body condition by pet owners frequently leads to under-recognition of overweight status, reducing adherence to veterinary recommendations [7]. Moreover, pharmacological options for treating obesity in pets are extremely limited, with no drugs currently approved specifically for weight loss in dogs or cats [2]. These gaps highlight the need for alternative, sustainable approaches that can be seamlessly integrated into daily feeding practices and that target multiple aspects of metabolic dysfunction relevant to veterinary patients.

In this context, plant-derived nutraceuticals and functional foods—particularly herbal extracts and herb-derived products—have attracted considerable attention as practical tools to support animal health and manage obesity-associated metabolic disorders. These preparations contain non-nutrient bioactive compounds with antioxidant, anti-inflammatory, lipid-lowering, and insulin-sensitizing activities and can be incorporated into daily diets, which is especially important in animals where long-term owner adherence is critical [6,8,9]. Within this broader class, herbal extracts and herb-derived products are increasingly promoted in veterinary medicine for their potential to preserve health status, modulate immune and metabolic pathways, and serve as complementary or alternative options to conventional medications [9]. Among the diverse phytochemicals present in herbal extracts, oleanane-type triterpene saponins have been reported to modulate inflammatory and metabolic signaling, suggesting the capacity to improve immunometabolic dysfunction and obesity-related pathology [10].

Previous research using rodent models has demonstrated that several botanical preparations, including green tea catechins, resveratrol, and curcumin, can effectively modulate key metabolic processes. In high-fat-diet-fed mice, such extracts have been shown to reduce body-weight gain and adiposity, improve glucose tolerance, normalize serum lipid profiles, and attenuate hepatic steatosis [11,12,13]. However, relatively few studies have combined long-term, dose-dependent evaluation of herbal extracts with comprehensive histopathological and molecular characterization while simultaneously linking extract composition to metabolic and inflammatory readouts in models that are informative for companion animals [14]. Such integrative designs are essential to support the rational development of herb-derived nutraceuticals and functional feed ingredients for veterinary use.

*Aster pekinensis* (AP) is a perennial herb belonging to the family Asteraceae. Members of the *Aster* genus, including *Aster tataricus*, are known to accumulate complex oleanane-type triterpenoid saponins and caffeoylquinic acid derivatives, which have been associated with anti-inflammatory, antidiabetic, hepatoprotective, and lipid-lowering activities [10,15,16,17]. Nevertheless, the ability of AP-derived phytochemicals to influence energy balance, glucose and lipid metabolism, and obesity-related liver injury has not been systematically tested in vivo. Defining the metabolite profile of AP and connecting it to metabolic outcomes in an established preclinical model would provide a rationale for considering AP as a herb-derived nutraceutical candidate for small animals.

To address this gap, we investigated whether a water extract of AP can ameliorate high-fat-diet (HFD)-induced obesity and metabolic disturbances in an established mouse model. Specifically, we evaluated the impact of chronic AP administration on body weight, regional adiposity, glucose homeostasis, serum lipid and liver enzyme profiles, and histologic and transcriptional markers of hepatic steatosis and inflammation in HFD-fed C57BL/6 mice.

In veterinary practice, nutritional and dietary management is central to the prevention and treatment of obesity, diabetes and NAFLD in dogs and cats, and there is growing interest in incorporating herbal extracts and herb-derived products into therapeutic and preventive diets as long-term, well-tolerated adjuncts to caloric restriction and exercise [18,19,20]. Although several plant extracts have demonstrated anti-obesity and hepatoprotective effects in high-fat-diet-fed C57BL/6 mice, most studies have focused on body weight, serum lipids and basic histology, and relatively few have simultaneously characterized systemic, hepatic and adipose-tissue outcomes using clinically relevant endpoints such as dynamic glucose tolerance tests, histological NAFLD scoring and transcriptional profiling of lipogenic and inflammatory pathways [21,22]. Establishing robust efficacy and mechanistic data in a well-validated HFD-induced obesity model in C57BL/6 mice is therefore a critical translational step before formulating AP–enriched functional feeds or nutraceutical products for dogs and cats [23,24].

Therefore, the present study aimed to characterize the phytochemical composition of a water extract of AP and to determine whether chronic oral administration of this extract attenuates HFD-induced obesity, NAFLD and insulin resistance in C57BL/6 mice through integrated assessment of systemic metabolic indices, hepatic histopathology and transcriptional markers that are relevant for the development of nutraceutical and functional-feed strategies for obese dogs and cats.

We therefore hypothesized that chronic oral administration of a water extract of AP would attenuate high-fat-diet-induced weight gain, adipocyte hypertrophy, non-alcoholic fatty liver disease and insulin resistance in C57BL/6 mice by downregulating hepatic de novo lipogenesis and inflammatory pathways, thereby providing a mechanistic preclinical rationale for developing AP-based nutraceutical formulations for dogs and cats.

## 2. Materials and Methods

### 2.1. Sample Extraction and Preparation

Aerial parts (leaves) of AP were collected from Goesan, South Korea. A total of 24 kg of AP leaves were extracted with 400 L of distilled water at 95 °C for 16 h. The resulting hot water extract was concentrated under reduced pressure to 5.0 °Brix and subsequently freeze-dried to yield 2.6 kg of powdered extract. For metabolite profiling, 100 mg of the freeze-dried powder was dissolved in 5 mL of distilled water and pre-cleared using Sep-Pak C18 cartridges (Waters, Milford, MA, USA). The cartridges were activated with 10 mL of methanol and equilibrated with 10 mL of distilled water. The sample was then loaded and sequentially eluted with 10 mL of distilled water and 10 mL of 80:20 (*v*/*v*) acetonitrile-distilled water. The 80% acetonitrile eluate was used for subsequent UPLC-ESI-QTOF-MS/MS analysis.

### 2.2. UPLC-ESI-QTOF-MS/MS Analysis

High-resolution electrospray ionization mass spectrometry (HR-ESI-MS) and metabolite profiling were performed using a quadrupole time-of-flight mass spectrometer (Xevo G2-XS QTOF, Waters, Manchester, UK) equipped with an ACQUITY UPLC system (Waters, Milford, MA, USA) and electrospray ionization (ESI) source. Separation was carried out using an ACQUITY UPLC HSS T3 column (1.8 μm, 2.1 × 100 mm, Waters) maintained at 40 °C. The mobile phase consisted of solvent A (water with 0.1% formic acid) and solvent B (acetonitrile with 0.1% formic acid). The gradient program was as follows: 5% B (0 min) → 5% B (2 min) → 10% B (5 min) → 25% B (15 min) → 40% B (33.5 min). The flow rate was set at 0.35 mL/min, and the injection volume was 1 μL. Mass spectrometric conditions were as follows: *m*/*z* 50–1200; scan time, 0.2 s; ionization mode, positive; capillary voltage, 2.5 kV; sampling cone voltage, 40 V; cone gas flow, 50 L/h; desolvation gas flow, 800 L/h; desolvation temperature, 400 °C; ion source temperature, 130 °C; collision energy, 6 eV (low) and 20–45 eV (high). Leucine-enkephalin was used as the lock mass at *m*/*z* 556.2771. Data were acquired and processed using MassLynx 4.1 software (Waters).

Prior to in vivo testing, the phytochemical composition of the AP extract was characterized by UPLC-ESI-QTOF-MS/MS using the method described in this section. Oleanane-type triterpenoid saponins and dicaffeoylquinic acids (DCQAs) were identified as the major constituents of the extract, providing a defined phytochemical background for the subsequent in vivo experiments.

### 2.3. Ethical Statement

All experimental procedures involving animals were approved by the Institutional Animal Care and Use Committee of Chonnam National University (CNU IACUC-YB-2024-73). Male C57BL/6 mice were housed under specific pathogen-free conditions in a temperature-controlled room (23 ± 2 °C), 50 ± 5% relative humidity, and a 12 h light/dark cycle, with 13–18 air changes per hour. Animals were provided standard rodent chow and water ad libitum and acclimated for one week prior to experimentation. All efforts were made to minimize animal suffering and the number of animals used.

### 2.4. Animal Study

Six-week-old male C57BL/6 mice were obtained from Samtako Bio Korea (Osan, Gyeonggi-do, Republic of Korea). This strain was selected because C57BL/6 mice reliably develop diet-induced obesity, hepatic steatosis and insulin resistance in response to long-term high-fat feeding and are widely used as a preclinical model of metabolic syndrome, as previously reported [23,24]. After a one-week acclimatization period, the mice were stratified by body weight and then randomly assigned to six groups (*n* = 6 per group) and subjected to a 12-week dietary intervention: (1) normal diet (ND), (2) high-fat diet with vehicle (HFD), (3) HFD + 10 mg/kg/day AP extract (AP 10), (4) HFD + 50 mg/kg/day AP extract (AP 50), (5) HFD + 100 mg/kg/day AP extract (AP 100), and (6) HFD + 200 mg/kg/day AP extract (AP 200). This experimental design corresponds to a preventive intervention model in which AP was administered concomitantly with the introduction of the HFD rather than after full establishment of obesity; the absence of baseline biochemical measurements prior to AP administration is acknowledged as a limitation in the Discussion. To minimize baseline differences among groups, animals were stratified into blocks according to body weight before randomization, resulting in comparable initial body weight distributions across all six groups.

The ND group received a purified low-fat control diet providing approximately 20% of total energy from protein, 10% from fat, and 70% from carbohydrates (~3.85 kcal/g), whereas the HFD groups were fed a very high-fat diet providing approximately 20% of total energy from protein, 60% from fat, and 20% from carbohydrates (~5.24 kcal/g), comparable to widely used diets for diet-induced obesity models [25,26]. The ND group received a standard chow diet composed of 20% protein, 5% fat, and 65% carbohydrates, whereas the HFD groups were fed a high-fat diet containing 19.7% protein, 60% fat, and 19.1% carbohydrates. The detailed macronutrient composition and energy density of the ND and HFD formulations are summarized in Table 1. AP extract or vehicle was dissolved in physiological saline and administered by oral gavage once daily throughout the study period. To ensure equivalent handling and stress across groups, mice in the ND and HFD control groups received the saline vehicle only, whereas AP-treated groups received the respective doses of AP dissolved in the same vehicle.

Body weight and food intake were recorded weekly. At the end of the experiment, the animals were anesthetized with isoflurane and euthanized. Major organs, including the liver, heart, kidneys, spleen, lungs, and adipose tissues, were harvested, weighed, and stored appropriately for subsequent histological and molecular analyses. Because blood was collected by cardiac puncture at the terminal time point, cardiac tissue was not used for histological or molecular evaluation in this study.

### 2.5. Histopathological Analysis

Liver and epididymal fat tissues were fixed in 10% neutral buffered formalin, dehydrated in ethanol, embedded in paraffin, and sectioned at 5 μm. Sections were stained with hematoxylin and eosin (H&E) and visualized under a light microscope (100× and 200× magnifications). Images were captured using the MoticEasyScan Infinity NFC 300 scanner (Motic, Xiamen, China). In epididymal white adipose tissue, adipocyte cross-sectional area was quantified from randomly selected fields using ImageJ software (version 1.8.0) to assess adipocyte hypertrophy. Qualitative evaluation of liver sections focused on the overall architecture, pattern of steatosis, hepatocellular ballooning and the presence of lobular inflammatory foci, while semi-quantitative NAFLD grading was performed as described in Section 2.6.

### 2.6. Liver Index and NAFLD Activity Score Analysis

Immediately after euthanasia, livers were excised, briefly rinsed in ice-cold PBS, blotted dry, and weighed to the nearest 0.01 g. The hepatic index (liver-to-body-weight ratio) was calculated as hepatic index (%) = (liver weight [g]/terminal body weight [g]) × 100, as described previously [27]. A representative portion of the left lateral lobe was fixed in 10% neutral-buffered formalin for histopathology, and the remaining tissue was snap-frozen in liquid nitrogen and stored at −80 °C for subsequent biochemical and molecular analyses.

NAFLD severity was evaluated using the NAFLD Activity Score (NAS), which semi-quantitatively grades macrovesicular steatosis (0–3), lobular inflammation (0–3), and hepatocellular ballooning (0–2) to yield a composite score ranging from 0 (no NAFLD) to 8 (severe disease) [28]. NAS scoring was applied to H&E-stained liver sections according to these criteria. For each animal, NAS was calculated as the mean of the scores assigned in eight randomly selected high-power fields (HPFs) per liver section; individual NAS values are therefore expressed as averaged scores, which can appear as non-integer (decimal) values.

### 2.7. Serum Biochemical Analysis

Whole blood was collected via cardiac puncture into heparinized tubes, incubated at room temperature for 30 min, and centrifuged at 3000 rpm for 15 min at 4 °C. The separated serum was stored at −80 °C until use. Serum levels of triglycerides (TG), total cholesterol (TC), high-density lipoprotein cholesterol (HDL-C), low-density lipoprotein cholesterol (LDL-C), alanine aminotransferase (ALT), aspartate aminotransferase (AST), and gamma-glutamyl transferase (GGT) were measured using standard enzymatic colorimetric methods on an automated chemistry analyzer (Dott 2000 Auto Chemistry Analyzer, MTD Diagnostics, San Marco Evangelista, Italy). Serum samples were stored at −80 °C for no longer than several weeks before analysis and were assayed after a single freeze–thaw cycle to minimize potential degradation.

### 2.8. Intraperitoneal Glucose Tolerance Testing (IPGTT) and Intraperitoneal Insulin Tolerance Testing (IPITT)

Mice underwent a 4 h morning fast for IPITT and an overnight fast of approximately 12 h for IPGTT. We recognize that this overnight fasting duration is longer than the 6 h fast commonly recommended for IPGTT in mice and may accentuate fasting-induced hypoglycemia; this methodological constraint is explicitly considered in the Limitations section of the Discussion.

For insulin quantification and HOMA calculations, an additional fasting blood sample (100 μL, tail vein) was collected at the indicated fasting time point (t = 0) into EDTA-K2 microtubes (Kangjian Medical, Jiangyan, China), centrifuged at 956× *g* for 20 min, and the resulting plasma was stored at −80 °C until insulin measurement.

The homeostatic model assessment indices were calculated as originally described [29]: HOMA-IR = [fasting glucose (mmol/L) × fasting insulin (μU/mL)]/22.5; HOMA-β = [20 × fasting insulin (μU/mL)]/[fasting glucose (mmol/L) − 3.5], expressed as %. Time-course glucose data for IPGTT and IPITT were analyses using a two-way repeated-measures ANOVA, as detailed in Section 2.10.

For both IPGTT and IPITT, area under the curve (AUC) for blood glucose was computed using the linear trapezoidal rule. Time-course data were analyzed as described in Section 2.10; AUC values were used for between-group comparisons as specified in the figure legends. For both IPGTT and IPITT, the AUC from 0 to 120 min was calculated using the linear trapezoidal rule, and AUC values are expressed as mmol/L × min.

### 2.9. Reverse Transcription qPCR (RT-qPCR)

Total RNA was extracted from liver tissue using TRIzol reagent (Invitrogen). One-step RT-qPCR was performed using the One-Step RT-qPCR Kit (Enzynomics, Daejeon, Republic of Korea) on a CFX Opus 96 Real-Time PCR System (Bio-Rad, Hercules, CA, USA). Reaction conditions were as follows: reverse transcription at 50 °C for 30 min, initial denaturation at 95 °C for 10 min, followed by 45 cycles of denaturation at 95 °C for 5 s and annealing/extension at 60 °C for 30 s. Gene expression was normalized to β-actin, and relative expression was calculated using the 2^−ΔΔCt^ method. Primer sequences are listed in Table 2.

### 2.10. Statistical Analysis

Data are expressed as mean ± standard deviation (SD). Longitudinal time-course data (weekly body weight, weekly food intake and IPGTT/IPITT glucose curves) were analyzed using two-way repeated-measures ANOVA with group, time and group × time interaction terms, followed by Tukey’s post hoc test for multiple comparisons at selected time points. Single time point outcomes and summary indices (e.g., organ weights, serum biochemistry, HOMA indices, NAFLD Activity Score, adipocyte cross-sectional area and AUC values for IPGTT and IPITT) were analyzed using one-way analysis of variance (ANOVA) followed by Tukey’s post hoc test. While all pairwise comparisons were conducted, primary focus was placed on comparisons between each treatment group and the HFD control group. Statis-tical analysis was performed using IBM SPSS Statistics version 29.0.2, with significance set at *p* < 0.05. Before applying parametric tests, we assessed normality of residuals using the Shapiro–Wilk test together with visual inspection of residual and Q–Q plots, and evaluated homogeneity of variances using Levene’s test. These diagnostics supported the use of ANOVA models for all reported outcomes.

## 3. Results

### 3.1. Identification of Oleanane-Type Saponins in AP Extract Using UPLC-ESI-QTOF-MS/MS

**Figure 1:**
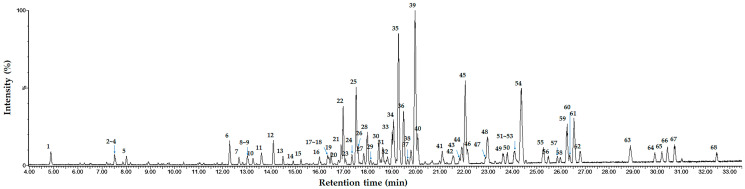
UPLC-ESI-QTOF-MS/MS TIC chromatogram of the AP extract.

**Figure 2:**
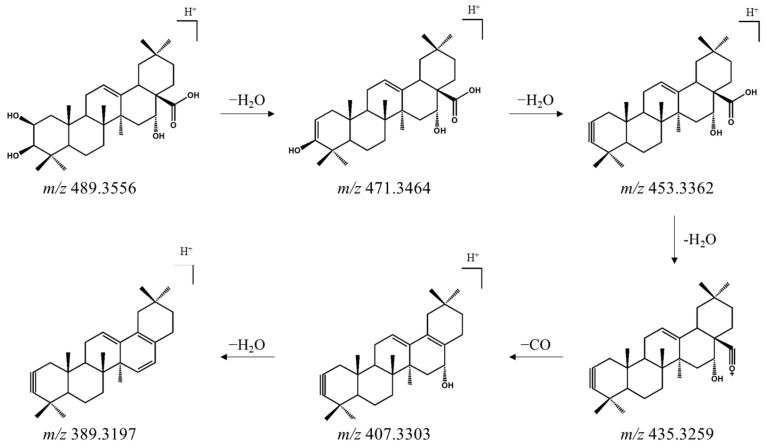
Proposed fragmentation pathway of 2,3,16-trihydroxyolean-12-en-28-oic acid in positive-ion mode.

The metabolites in AP extract were characterized by UPLC-ESI-QTOF-MS/MS analysis, revealing a complex chromatographic profile dominated by triterpenoid saponins (Figure 1, Table 3). MS/MS fragmentation patterns and HR-ESI-MS data of the compounds exhibited characteristic neutral losses of glucose, rhamnose, and xylose from [M + Na]^+^ and [M + H]^+^ precursor ions, consistent with oleanane-type triterpenoid saponins. The key aglycone, exhibiting sequential neutral losses of H_2_O and CO, was identified as 2,3,16-trihydroxyolean-12-en-28-oic acid (Figure 2), an oleanane scaffold characteristic of saponins from *Aster* species. On this basis, we tentatively assigned saponins derived from 2,3,16-trihydroxyolean-12-en-28-oic acid—featuring variations in hydroxylation and dehydroxylation, dehydrogenation, carboxylation, and lactone formation—as major constituents of the AP extract. While the observed MS/MS fragmentation patterns allow annotation of these saponin families, isolation of individual compounds followed by NMR-based structural elucidation will be required to define sugar epimers and linkage positions and to pinpoint the specific bioactive principles.

### 3.2. AP Extract Reduces Body-Weight Gain and Improves Food Efficiency in C57BL/6 Mice with HFD-Induced Obesity

**Figure 3:**
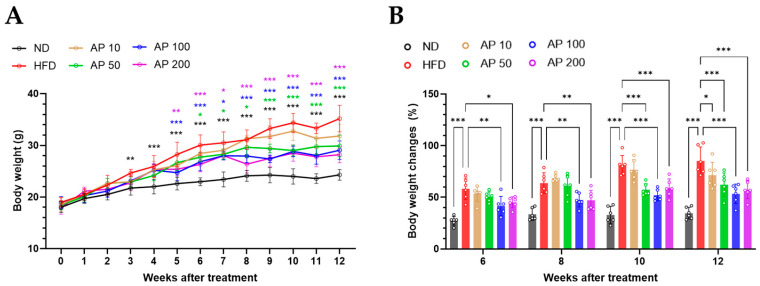
Effects of AP extract on body weight in HFD-induced obese mice over 12 weeks. (**A**) Cumulative body-weight gain over 12 weeks (mean ± SD, *n* = 6/group). Statistics: one-way ANOVA with Dunnett’s multiple comparisons versus the HFD group at each time point. (**B**) Baseline-normalized body-weight change relative to each group’s week-0 mean at weeks 6, 8, 10, and 12. Statistics: (**A**,**B**) two-way repeated-measures ANOVA with group, time and group × time interaction, followed by Tukey’s post hoc test for between-group comparisons at selected time points; Significance: (**A**) * *p* < 0.05, ** *p* < 0.01, *** *p* < 0.001 versus HFD; (**B**) * *p* < 0.05, ** *p* < 0.01, *** *p* < 0.001 versus the indicated comparator within the panel.

Body weight was monitored weekly over 12 weeks. As expected, HFD-fed mice showed progressive and sustained weight gain, resulting in markedly higher body weight than ND controls by the end of the study (Figure 3A). AP treatment attenuated HFD-induced weight gain in a dose-dependent manner. AP 100 mg/kg/day produced the most pronounced effect, with body weight diverging from the HFD group from the mid-phase of the experiment and remaining significantly lower through week 12. AP 50 mg/kg/day also significantly reduced HFD-induced weight gain, although with a more modest magnitude, whereas AP 10 mg/kg/day exerted only minor effects. AP 200 mg/kg/day did not consistently outperform AP 100 mg/kg/day, indicating a plateau in efficacy at higher doses.

Baseline-normalized body-weight change analysis yielded a similar pattern (Figure 3B). Compared with HFD controls, AP 50–100 mg/kg/day significantly attenuated cumulative increases in body weight at multiple prespecified time points, consistent with a threshold-like effective dose window between 50 and 100 mg/kg/day, with maintained but not incrementally greater efficacy at 200 mg/kg/day.

**Figure 4:**
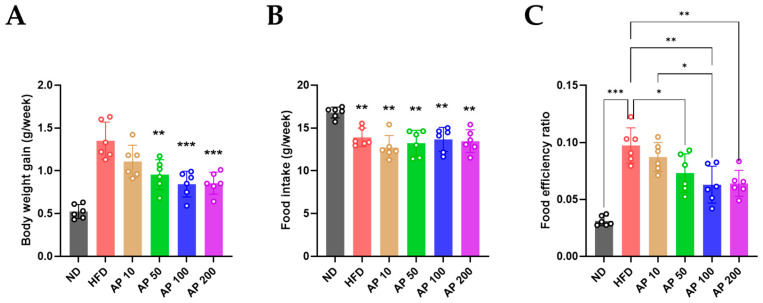
Effects of AP extract on body weight gain, food intake, and food efficiency ratio (FER) in HFD-induced obese mice. (**A**) Weekly absolute body weight (g) over 12 weeks. (**B**) Weekly food intake (g) over 12 weeks. (**C**) FER per group. Data are presented as mean ± SD (*n* = 6/group for all panels). Statistics: (**A**,**B**) two-way repeated-measures ANOVA with group, time and group × time interaction, followed by Tukey’s post hoc test; (**C**) one-way ANOVA with Tukey’s post hoc test for between-group comparisons of cumulative feed efficiency ratio. Significance: * *p* < 0.05, ** *p* < 0.01, *** *p* < 0.001.

After 12 weeks, cumulative body-weight gain was significantly increased in HFD-fed mice relative to ND controls (Figure 4A). AP 50–200 mg/kg/day significantly reduced cumulative gain compared with HFD, whereas AP 10 mg/kg/day again showed limited effect. Food intake (g/mouse/week) did not differ substantially among HFD and AP-treated groups and remained lower than in ND animals, reflecting the higher energy density of the HFD (Figure 4B). In contrast, the food-efficiency ratio (body-weight gain per unit of food intake) was significantly elevated in HFD-fed mice compared with ND and was dose-dependently reduced by AP 50–200 mg/kg/day (Figure 4C), indicating that AP improved metabolic handling of dietary calories rather than simply reducing food consumption.

### 3.3. AP Extract Reduces Fat Accumulation in C57BL/6 Mice with HFD-Induced Obesity

**Figure 5:**
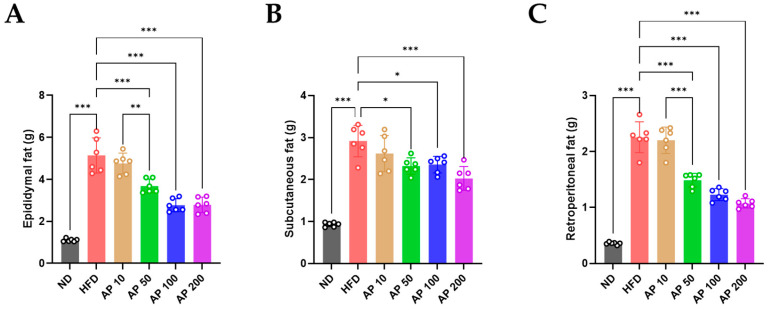
Effects of AP extract on adipose-tissue mass in HFD-induced obese mice. (**A**) Epididymal fat, (**B**) subcutaneous fat, and (**C**) retroperitoneal fat weights (g). Data: mean ± SD, *n* = 6 per group. Statistics: one-way ANOVA with Tukey’s all-pairwise comparisons among all groups. Significance: * *p* < 0.05, ** *p* < 0.01, *** *p* < 0.001.

Consistent with the body-weight data, adiposity was markedly increased in HFD-fed mice. Epididymal, subcutaneous, and retroperitoneal WAT depots were significantly heavier in HFD animals than in ND controls (Figure 5), confirming robust diet-induced adiposity. AP treatment reduced WAT mass in a dose-dependent manner. AP 50–200 mg/kg/day significantly decreased the weight of all three fat depots compared with HFD, whereas AP 10 mg/kg/day produced only minor or non-significant changes.

### 3.4. AP Extract Attenuates Adipocyte Hypertrophy in Epididymal WAT

**Figure 6:**
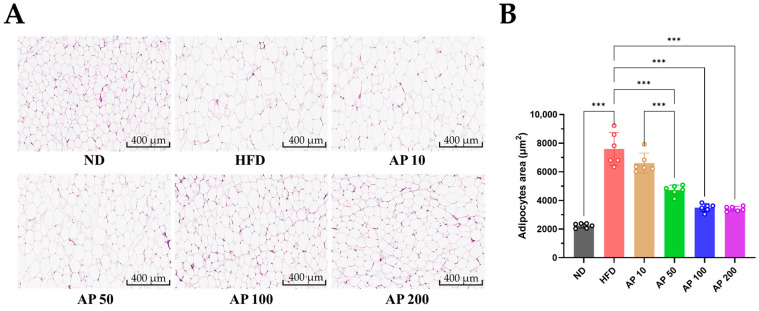
Effect of AP extract on adipocyte hypertrophy in epididymal white adipose tissue (WAT) of HFD-induced obese mice. (**A**) Representative H&E-stained sections of WAT showing adipocyte morphology. HFD-fed obese mice exhibited hypertrophic adipocytes, whereas extract-treated mice showed markedly reduced adipocyte size (original magnification 200×, scale bar = 100 μm). (**B**) Quantification of adipocyte area based on randomly selected fields analyzed using ImageJ software. Mice were orally administered the extract daily for 12 weeks. Data are presented as mean ± SD (*n* = 6/group). Statistics: one-way ANOVA with Tukey’s all-pairwise comparisons among all groups. Significance: *** *p* < 0.001.

Histological analysis of epididymal WAT by H&E staining demonstrated marked adipocyte hypertrophy in HFD-fed mice, with enlarged adipocyte cross-sectional areas and reduced cellularity per field relative to ND controls (Figure 6A). Quantitative morphometry confirmed a significant rightward shift in the adipocyte-size distribution in HFD versus ND (Figure 6B). AP 50–200 mg/kg/day reduced adipocyte size toward ND values, decreasing mean cross-sectional area and partially restoring the proportion of smaller adipocytes, whereas AP 10 mg/kg/day showed only a modest effect. These findings indicate that AP not only limits fat-pad expansion but also counteracts adipocyte hypertrophy, a key morphological feature of metabolically unhealthy obesity.

### 3.5. AP Improves Blood Glucose and Blood Lipids in HFD-Induced Obese Mice

**Figure 7:**
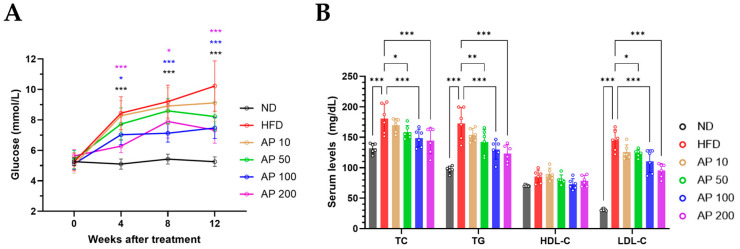
Effects of AP extract on serum glucose and lipid profiles in HFD-induced obese mice. (**A**) Fasting blood glucose (FBG) after 12 weeks. (**B**) Lipid panel: triglycerides (TG), total cholesterol (TC), HDL-cholesterol (HDL-C), LDL-cholesterol (LDL-C), and the LDL-C/HDL-C ratio as an atherogenic index. Mice received daily oral AP for 12 weeks. Data are presented as mean ± SD (*n* = 6/group). Statistics: (**A**) one-way ANOVA with Dunnett’s multiple comparisons versus the HFD group; (**B**) one-way ANOVA with Tukey’s all-pairwise comparisons among all groups. Significance: * *p* < 0.05, ** *p* < 0.01, *** *p* < 0.001 (versus the indicated comparator within each panel).

After 12 weeks, HFD feeding significantly increased fasting blood glucose compared with ND controls and adversely affected circulating lipid profiles, as reflected by higher serum TG, TC, and LDL-C (Figure 7). AP 50–200 mg/kg/day significantly lowered fasting glucose and reduced TG, TC, and LDL-C in a dose-responsive manner relative to HFD, whereas AP 10 mg/kg/day exerted limited effects. High-density lipoprotein cholesterol (HDL-C) remained largely unchanged among groups. Because the LDL-C/HDL-C ratio is widely used as a clinically meaningful indicator of atherogenic risk, we also evaluated this composite index and found that AP 50–200 mg/kg/day significantly improved LDL-C/HDL-C ratios versus HFD, indicating a shift toward a more favorable lipid profile.

### 3.6. AP Attenuates HFD-Induced Hepatomegaly, NAFLD, and Liver Injury

**Figure 8:**
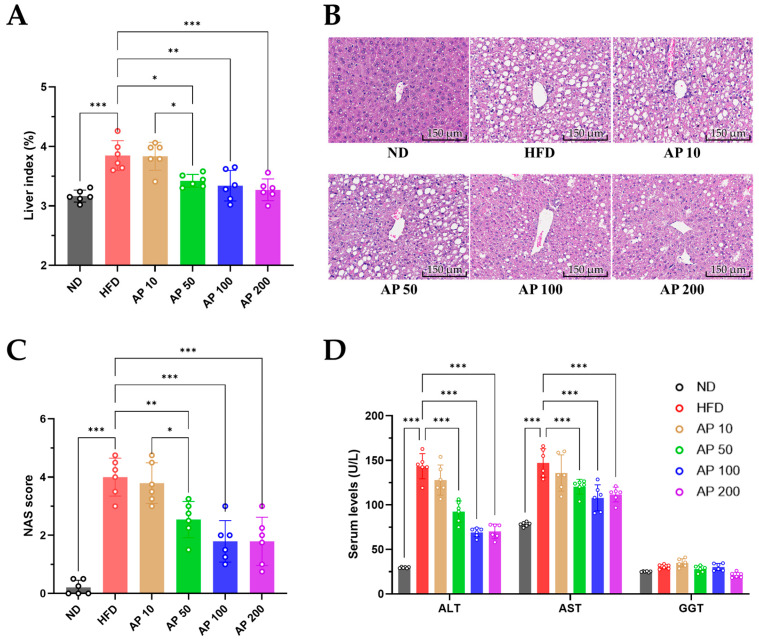
Effects of AP on hepatic endpoints related to HFD-induced NAFLD in mice. (**A**) Hepatic index (%). (**B**) Representative H&E-stained liver sections showing macrovesicular steatosis, hepatocellular ballooning, and lobular inflammatory foci in each group (original magnification 400×, scale bar = 100 μm). (**C**) NAFLD Activity Score (NAS), calculated from steatosis, lobular inflammation, and ballooning degeneration. (**D**) Serum activities of liver injury markers ALT, AST, and GGT. Data for panels (**A**,**C**,**D**) are presented as mean ± SD (*n* = 6/group). Statistics: one-way ANOVA with Tukey’s all-pairwise comparisons among all groups. Significance: * *p* < 0.05, ** *p* < 0.01, *** *p* < 0.001 (versus the indicated comparator within each panel).

Chronic HFD feeding is known to induce NAFLD-like hepatic changes in C57BL/6 mice, a pattern that was recapitulated in our model. Relative to ND controls, HFD-fed mice displayed hepatomegaly, evidenced by a significantly higher hepatic index (Figure 8A). Histologically, HFD livers showed prominent macrovesicular steatosis, hepatocellular ballooning, and lobular inflammatory foci on H&E-stained sections (Figure 8B). Consistent with these qualitative findings, NAS incorporating steatosis, lobular inflammation, and ballooning degeneration were markedly higher in HFD-fed mice than in ND controls, and AP 50–200 mg/kg/day significantly lowered composite NAS compared with HFD (Figure 8C). Concordantly, serum ALT and AST activities were elevated in HFD versus ND and were significantly decreased by AP 50–200 mg/kg/day (Figure 8D), indicating that AP mitigates HFD-induced hepatomegaly, NAFLD severity, and liver injury in this model.

### 3.7. AP Improves IPGTT/IPITT and Modulates Hepatic De Novo Lipogenesis and Inflammation

**Figure 9:**
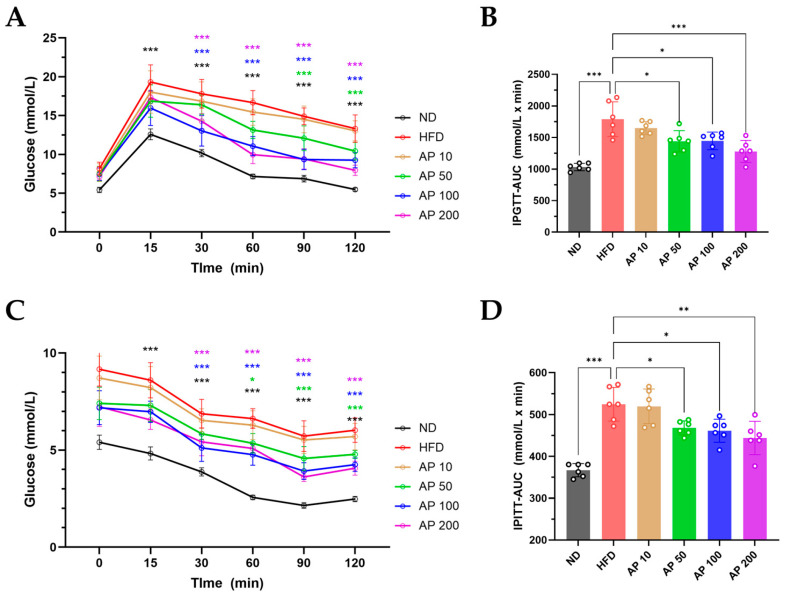
Effects of AP on glucose tolerance and insulin sensitivity. (**A**) IPGTT: blood glucose levels measured at 0, 30, 60, and 120 min after intraperitoneal glucose (2 g/kg). (**B**) IPGTT: area under the curve (AUC) for blood glucose over 120 min. (**C**) IPITT: blood glucose levels measured at 0, 15, 30, 45, 60, and 120 min after intraperitoneal insulin (0.5 U/kg). (**D**) IPITT: AUC for blood glucose over 120 min. Data are presented as mean ± SEM (*n* = 6/group). Statistics: (**A**,**C**) two-way repeated-measures ANOVA with group, time and group × time interaction, followed by Tukey’s post hoc test; (**B**,**D**) one-way ANOVA with Tukey’s post hoc test for comparisons of AUC values. Significance: * *p* < 0.05, ** *p* < 0.01, *** *p* < 0.001 (versus the indicated comparator within each panel).

Glucose handling and insulin responsiveness were assessed by IPGTT and IPITT at the end of the study (Figure 9). In the IPGTT, HFD-fed mice exhibited impaired glucose tolerance, with higher blood-glucose excursions and increased AUC compared with ND controls. AP 50–200 mg/kg/day significantly reduced peak glycemia and lowered IPGTT AUC in a dose-responsive manner, whereas AP 10 mg/kg/day showed little or no effect (Figure 9A,B). In the IPITT, HFD-fed mice displayed blunted glucose-lowering responses to exogenous insulin, whereas AP 50–200 mg/kg/day enhanced the decline in blood glucose and improved the corresponding AUC relative to HFD, again with evidence of a plateau between 100 and 200 mg/kg/day (Figure 9C,D). These functional readouts indicate that AP improves whole-body glucose tolerance and insulin sensitivity in HFD-induced obese mice.

**Figure 10:**
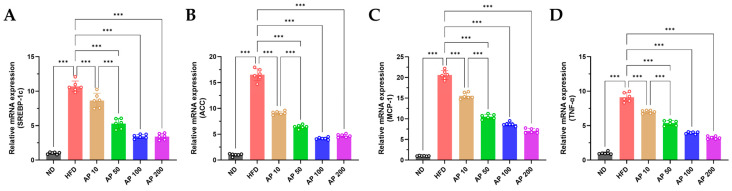
Effects of AP on hepatic gene expression after 12 weeks. RT-qPCR of (**A**) SREBP-1c, (**B**) ACC, (**C**) MCP-1, and (**D**) TNF-α in liver tissue. Expression is shown as fold-change versus ND (2^−ΔΔCt^, normalized to a housekeeping gene). Data are mean ± SD (*n* = 6/group). Statistics: one-way ANOVA with Tukey’s all-pairwise comparisons among all groups. Significance: *** *p* < 0.001 (versus the indicated comparator within each panel).

At the molecular level, HFD feeding significantly up-regulated hepatic SREBP-1c and its downstream lipogenic enzyme ACC, while also increasing expression of the pro-inflammatory mediators MCP-1 and TNF-α, consistent with heightened hepatic metabolic and inflammatory stress (Figure 10). AP 50–200 mg/kg/day reduced SREBP-1c and ACC transcripts toward ND levels and concomitantly suppressed MCP-1 and TNF-α expression. These gene-expression changes parallel group-wise differences observed in hepatic index, NAFLD scores, and serum transaminases (Figure 8) and support a broad attenuation of HFD-induced hepatic metabolic and inflammatory stress under AP treatment.

## 4. Discussion

In the present study, 12 weeks of very high-fat feeding in C57BL/6 mice produced a robust obese–NAFLD phenotype characterized by increased terminal body weight and WAT depot masses, marked hypertrophy of epididymal adipocytes, fasting hyperglycemia, impaired IPGTT/IPITT responses, dyslipidemia and elevated hepatic index, NAFLD activity scores and serum transaminases in HFD controls compared with ND animals. The pattern of steatosis, lobular inflammation, and hepatocellular ballooning observed in HFD-fed C57BL/6 mice in our study is in line with previous characterizations of high fat diet-induced NAFLD in this strain [23,32]. Together with prior work, these findings support the current paradigm in which chronic caloric excess drives adipocyte hypertrophy, inflammation, and ectopic lipid deposition, ultimately promoting hepatic lipotoxicity and progression from simple steatosis to steatohepatitis and related metabolic complications [33,34,35,36].

Against this background, AP extract exerted coordinated systemic and organ-level effects that counteracted multiple components of the HFD-induced phenotype. The LDL-C/HDL-C ratio is widely used as a surrogate marker of atherogenic risk in cardiovascular prevention [37], and the reduction in this ratio by AP further supports a favorable cardiometabolic profile. AP at 50–200 mg/kg/day attenuated body-weight gain and WAT enlargement despite similar cumulative food intake, reduced the proportion of markedly hypertrophied adipocytes in epididymal fat, improved fasting glucose and HOMA indices and lowered serum triglycerides and liver enzymes compared with HFD controls. These benefits were accompanied by reductions in hepatic index and NAFLD scores, with less macrovesicular steatosis, ballooning and lobular inflammation, and downregulation of hepatic Srebp-1c, Acc1, Mcp-1, and Tnf-α, indicating that AP mitigates adipocyte hypertrophy and hepatic lipotoxicity in HFD-fed mice.

The dissociation between reduced body-weight gain and unchanged weekly food intake in AP-treated mice suggests that AP primarily modulated feed efficiency and nutrient handling rather than suppressing appetite. This pattern is compatible with several non-mutually exclusive mechanisms, including increased whole-body energy expenditure (e.g., thermogenesis or physical activity), reduced intestinal fat absorption and intrinsic changes in adipocyte lipid storage and turnover [38,39]. Similar dissociations between food intake and weight gain have been reported for other botanical preparations, such as green tea catechins, resveratrol and curcumin, which reduced body-weight gain and WAT mass while improving glucose tolerance, serum lipid profiles and hepatic steatosis in HFD-fed C57BL/6 mice [11,18,22]. In companion animals, where slower metabolism, resistance to caloric restriction and limited owner adherence to weight-management plans are common, such a multi-target adjunct that improves metabolic health without further reducing food intake could be particularly attractive [40,41,42].

A second key observation is that AP reduced the proportion of hypertrophied adipocytes in epididymal WAT, which was associated with lower WAT mass and improved systemic metabolic indices. Adipocyte hypertrophy is strongly linked to local hypoxia, crown-like structure formation, M1-polarized macrophage infiltration and secretion of pro-inflammatory adipokines that drive insulin resistance in adipose tissue, liver and skeletal muscle [36,43]. By limiting adipocyte enlargement, AP may reduce adipose-tissue inflammation and lipolytic flux of fatty acids to the liver, which is consistent with the observed downregulation of hepatic SREBP1C and ACC1 and the improvement in NAFLD histology and liver enzymes.

Consistent with previous phytochemical studies of *Aster* species [15,16,17,44], UPLC-ESI-QTOF-MS/MS profiling indicated that the AP extract used in this study is enriched in oleanane-type triterpenoid saponins and DCQAs, providing a plausible phytochemical basis for these metabolic and anti-inflammatory actions. Oleanane-type triterpenoid saponins from several plant species have been reported to activate AMPK and PPARα, suppress NF-κB signaling and downregulate lipogenic transcription factors such as SREBP-1c and ACC, thereby reducing adiposity and hepatic triglyceride accumulation in HFD-fed rodents [45,46,47]. DCQAs and related caffeoylquinic acids can activate Nrf2-dependent antioxidant pathways and inhibit NF-κB-driven inflammatory responses, and they undergo extensive transformation by the gut microbiota, which can further modulate host metabolic and immune homeostasis [48,49,50].

Collectively, these data suggest that AP-derived oleanane-type triterpenoid saponins and DCQAs act via AMPK/PPARα and Nrf2/NF-κB axes to reduce de novo lipogenesis, enhance mitochondrial fatty-acid oxidation and attenuate inflammatory signaling in adipose tissue and liver. Future studies should test this mechanism by quantifying phospho-AMPK, nuclear NF-κB p65, NRF2 and downstream targets, as well as IRS-1/IRS-2/Akt/GSK3β signaling in adipose tissue and liver [51,52,53].

The parallel improvement in IPGTT/IPITT curves, HOMA indices and hepatic steatosis under AP treatment suggests coordinated amelioration of both hepatic and peripheral insulin resistance. We propose that improved insulin signaling, driven by reduced hepatic lipotoxicity and adipose inflammation, underlies these systemic glycemic benefits [34,54,55]. This integrated view links the systemic glycemic benefits of AP to its effects on WAT and liver, providing a mechanistic framework that is relevant to companion animals, in which NAFLD and type 2 diabetes frequently coexist with obesity.

In terms of dose selection, AP at 50 and 100 mg/kg/day consistently produced metabolic benefits, whereas 10 mg/kg/day was largely ineffective and 200 mg/kg/day did not offer clear additional advantages beyond 100 mg/kg/day. This non-linear pattern is compatible with the pharmacokinetic behavior of saponins and polyphenolic acids, which often display low and saturable oral absorption, extensive first-pass metabolism and microbiota-dependent biotransformation [56,57,58,59]. For translational purposes, we adopted a standard body-surface-area (BSA)-based interspecies dose conversion, in which the equivalent dose in a target species is calculated as target dose (mg/kg) = mouse dose (mg/kg) × (*Km*_mouse/*Km*_target), where *Km* denotes the species-specific correction factor [60,61]. Using *Km* values of 3 for mice and 20 for dogs, the 50–100 mg/kg/day doses employed in this study correspond to approximately 7.5–15 mg/kg/day in dogs; assuming a *Km* of 13 for cats, the same mouse doses would translate to approximately 11.5–23 mg/kg/day (reported here as ~8–15 mg/kg/day for dogs and ~12–23 mg/kg/day for cats). These estimates provide an initial quantitative framework for designing pilot safety and efficacy studies in target species; however, they should be regarded only as a starting point, and species-specific pharmacokinetic, safety, and palatability studies will be essential to define clinically applicable dosing regimens for dogs and cats [60,61].

Several limitations warrant consideration when extrapolating these findings. First, only male C57BL/6 mice and a single HFD formulation were studied, so the influence of sex, genetic background and dietary composition on the response to AP remains unknown, and we did not evaluate efficacy in animals with established severe obesity [62,63]. Second, we did not perform a formal multivariate integrative analysis (e.g., principal component analysis) across all metabolic, histologic, and gene-expression endpoints; although such methods can reveal coordinated patterns of response, the relatively small group sizes and large number of variables in this exploratory study raised concerns about potential overfitting and unstable component structure, so we focused on prespecified univariate analyses and summary indices, and future studies with larger cohorts will be designed to incorporate multivariate approaches to better capture the joint structure of the dataset [64,65]. Third, hepatic outcomes were evaluated at the mRNA level without complementary protein or signaling measurements, which limits mechanistic certainty regarding the involvement of AMPK/PPARα and Nrf2/NF-κB pathways. Fourth, although UPLC-ESI-QTOF-MS/MS identified major saponin and DCQA families, individual bioactive compounds were not fully quantified or isolated, and pharmacokinetic data are lacking, so the precise exposure–response relationships and active species remain to be defined. Moreover, only a limited number of phytochemical and pharmacological investigations of AP and closely related *Aster* species have been reported, predominantly from East Asian populations [15,16,17,44], so additional work in diverse geographic settings will be needed to confirm these findings and to develop standardized AP preparations suitable for broader translational use. Fifth, we did not analyze gut microbiota composition, even though DCQAs and saponins are extensively metabolized by intestinal microbes and can modulate microbial ecology, which may contribute substantially to AP’s systemic actions. Sixth, the study followed a preventive design, with AP introduced at the same time as HFD and without baseline biochemical measurements; effects in animals with established obesity, diabetes or NAFLD were not addressed. In addition, we did not include a pharmacological positive control (e.g., orlistat) in the experimental design; although several studies of plant-derived interventions in high-fat diet models have incorporated such comparators [64,66], our primary objective in the present work was to delineate the dose–response profile and mechanistic effects of AP itself rather than to perform head-to-head comparisons with an approved anti-obesity drug. Seventh, because terminal blood sampling was performed by cardiac puncture, we did not carry out histological or molecular analyses of the heart, and potential direct cardiac effects of AP were not evaluated in this study. Eighth, IPGTT was performed after an overnight fast that exceeds current recommendations for mice, which may exaggerate fasting-induced hypoglycemia; this protocol is now explicitly acknowledged as a methodological limitation of the glycemic outcomes and should be refined to a shorter fasting period in future experiments. Finally, AP was administered once daily by oral gavage rather than as a dietary component or treat; while this ensured accurate dosing, it does not fully reflect how nutraceuticals are typically delivered to dogs and cats, and future work should evaluate AP formulated directly into diets or snacks with long-term safety monitoring.

Despite these caveats, our data show that AP simultaneously targets adiposity, adipocyte morphology, hepatic steatosis and systemic glucose regulation in a well-established HFD-induced obesity model, providing a coherent mechanistic rationale for developing AP-enriched functional diets or nutraceutical products aimed at managing obesity-associated metabolic disorders in dogs and cats. These preclinical results therefore lay the groundwork for controlled translational studies in companion animals that integrate metabolic endpoints, histopathology and extended safety outcomes over clinically relevant treatment periods.

## 5. Conclusions

In a high-fat-diet-induced obesity and NAFLD mouse model, chronic administration of an aqueous AP extract attenuated adiposity, improved systemic glycemic control, and ameliorated hepatic steatosis and injury. By simultaneously targeting adipose tissue, liver, and metabolic regulation, AP emerges as a promising botanical candidate for development as a nutraceutical adjunct to weight-management strategies in obese companion animals. These findings provide a mechanistic and translational framework for AP–enriched functional diets or supplements, while underscoring the need for future studies to define species-appropriate dosing, pharmacokinetics, long-term safety, and clinical efficacy in dogs and cats.

## Figures and Tables

**Table 1:** Approximate macronutrient composition and energy density of the ND and HFD used in the mouse study.

Parameter	ND	HFD
Protein (% of total energy)	20	20
Fat (% of total energy)	10	60
Carbohydrate (% of total energy)	70	20
Energy density (kcal/g)	3.85	5.24

Values are expressed as percentage of total metabolizable energy (kcal). The composition and energy density are comparable to commonly used purified rodent diets for diet-induced obesity models [25,26].

**Table 2:** Primer Sequences Used for RT-qPCR.

Name	Forward (5′ → 3′)	Reverse (5′ → 3′)	Source
ACC1	TTCAGTTCATGCTGCCCACA	AGGTTGGAGGCAAAGGACAT	[30]
SREBP-1c	AAGACAGATGCAGGAGCCAC	CCTCCACTCACCAGGGTCT	[30]
TNF-α	CCCACGTCGTAGCAAACCA	CTTTGAGATCCATGCCGTTGG	[30]
MCP-1	TCACCAGCAAGATGATCCCA	GAGCTTGGTGACAAAAACTACA	[30]
β-actin	AGCCTTCCTTCTTGGGTATGG	CACTTGCGGTGCACGATGGAG	[31]

Primers designed for this study using the NCBI Primer Design tool. Abbreviations: ACC, acetyl-CoA carboxylase; SREBP-1c, sterol regulatory element-binding protein-1c; MCP-1, monocyte chemoattractant protein-1; TNF-α, tumor necrosis factor-α.

**Table 3:** Chemical library of the AP extract.

No.	*t_R_* (min)	Mode of Ionization	Measured Mass (Da)	Calculated Mass (Da)	Mass Error (ppm)	Molecular Formula	Fragment Ions (*m*/*z*)	Predicted Compounds
1	4.88	[M + Na]^+^	551.1940	551.1952	−0.3	C_21_H_36_O_15_	483.1490, 323.1076	Unknown
2	7.51	[M + Na]^+^	771.2526	771.2535	−0.3	C_29_H_48_O_22_	443.3215	Unknown
3	7.52	[M + Na]^+^	425.1437	425.1424	−0.2	C_18_H_26_O_10_	343.1753	Unknown
4	7.54	[M + Na]^+^	409.1836	409.1838	1.4	C_19_H_30_O_8_	207.1388	Vomifoliol glucoside
5	8.01	[M + Na]^+^	409.1831	409.1838	0.0	C_19_H_30_O_8_	207.1387	Vomifoliol glucoside
6	12.28	[M + H]^+^	517.1342	517.1346	0.8	C_25_H_24_O_12_	499.1231, 163.0396	3,4-Dicaffeoylquinic acid
7	12.67	[M + Na]^+^	539.1157	539.1165	0.4	C_25_H_24_O_12_	499.1237, 163.0396	3,5-Dicaffeoylquinic acid
8	13.01	[M + Na]^+^	605.1471	605.1482	−0.9	C_26_H_30_O_15_	343.1026	Unknown
9	13.05	[M + Na]^+^	527.2460	527.2468	−0.2	C_24_H_40_O_11_	319.2268	Unknown
10	13.26	[M + H]^+^	479.1187	479.1190	−1.3	C_22_H_22_O_12_	317.0656	Isorhamnetin-3-glucoside
11	13.60	[M + H]^+^	517.1348	517.1346	−0.4	C_25_H_24_O_12_	499.1243, 163.0395	4,5-Dicaffeoylquinic acid
12	14.10	[M + Na]^+^	681.2338	681.2336	3.7	C_30_H_42_O_16_	601.2844, 533.1143, 517.1438, 461.1841, 397.1255	Unknown
13	14.50	[M + Na]^+^	601.2838	601.2836	0.3	C_27_H_46_O_13_	421.2197, 309.1273	Unknown
14	14.92	[M + H]^+^	1269.6136	1269.6116	1.6	C_59_H_96_O_29_	1107.5569, 945.5045, 813.4557, 667.4038, 487.3434	2,3,6,16-Tetrahydroxyolean-12-en-28-oic acid + 3Glc + Rha + Xyl
15	15.24	[M + Na]^+^	1261.5823	1261.5829	−0.5	C_58_H_94_O_28_	1099.5304, 487.3423	2,3,6,16-Tetrahydroxyolean-12-en-28-oic acid + 3Glc+ Rha + Xyl
16	16.00	[M + Na]^+^	1437.6548	1437.6514	2.4	C_65_H_106_O_33_	813.4620, 651.4080, 453.3378	2,3,16-Trihydroxyolean-12-en-28-oic acid + 4Glc + Rha+ Xyl
17	16.34	[M + H]^+^	1385.6667	1385.6589	5.6	C_64_H_104_O_32_	783.4531, 453.3374	2,3,16-Trihydroxyolean-12-en-28-oic acid + 3Glc + Rha + 2Xyl
18	16.39	[M + H]^+^	1385.6633	1385.6589	3.2	C_64_H_104_O_32_	783.4539, 453.3339	2,3,16-Trihydroxyolean-12-en-28-oic acid + 3Glc + Rha + 2Xyl
19	16.48	[M + H]^+^	1253.6161	1253.6166	−0.4	C_59_H_96_O_28_	1091.5657, 651.4111, 453.3368	2,3,16-Trihydroxyolean-12-en-28-oic acid + 3Glc + Rha + Xyl
20	16.80	[M + H]^+^	1355.6559	1355.6483	5.6	C_63_H_102_O_31_	1223.6074, 929.5042, 783.4551, 651.4108, 453.3361	2,3,16-Trihydroxyolean-12-en-28-oic acid + 2Glc + Rha + 3Xyl
21	16.90	[M + H]^+^	1223.6062	1223.6061	0.1	C_58_H_94_O_27_	1061.5507, 929.5118, 797.4664, 783.4521, 651.4119, 453.3367	2,3,16-Trihydroxyolean-12-en-28-oic acid + 2Glc + Rha + 2Xyl
22	16.98	[M + H]^+^	1253.6179	1256.6177	1.0	C_59_H_96_O_28_	1091.5616, 929.5111, 797.4672, 651.4097, 453.3361	2,3,16-Trihydroxyolean-12-en-28-oic acid + 3Glc + Rha + Xyl
23	17.09	[M + H]^+^	1355.6532	1355.6483	3.6	C_63_H_102_O_31_	1223.6051, 1077.5493, 929.5161, 783.4519, 651.4115, 453.3372	2,3,16-Trihydroxyolean-12-en-28-oic acid + 2Glc + Rha + 3Xyl
24	17.35	[M + H]^+^	1267.5990	1267.5959	2.4	C_59_H_94_O_29_	1105.5425, 943.4988, 453.3363	Triterpenoid peantaglycoside
25	17.52	[M + H]^+^	1223.6061	1223.6061	0.0	C_58_H_94_O_27_	1091.5635, 929.5109, 797.4680, 651.4106, 453.3366	2,3,16-Trihydroxyolean-12-en-28-oic acid + 2Glc + Rha + 2Xyl
26	17.58	[M + H]^+^	1091.5642	1091.5638	0.4	C_53_H_86_O_23_	929.5074, 797.4469, 651.4102, 453.3365	2,3,16-Trihydroxyolean-12-en-28-oic acid + 2Glc + Rha + Xyl
27	17.84	[M + H]^+^	1237.5847	1237.5853	−0.5	C_58_H_92_O_28_	1075.5381, 959.4803, 503.3332, 485.3268	2,3-Dihydroxyolean-12-en-23,28-dioic acid + 2Glc + Rha + 2Xyl
28	17.99	[M + Na]^+^	965.4346	965.4335	1.1	C_46_H_70_O_20_	517.3152, 499.3055	2,3,16,21-Tetrahydroxyolean-12-en-23,28-dioic acid 28,21-lactone+ Glc + 2Xyl
29	18.12	[M + Na]^+^	1405.6241	1405.6252	−0.8	C_64_H_102_O_32_	487.3412, 469.3289	2,3,16-Trihydroxyolean-11,13-dien-28-oic acid + 3Glc + Rha + 2Xyl
30	18.46	[M + Na]^+^	1113.5409	1113.5458	−4.4	C_53_H_86_O_23_	929.5071, 767.4554, 635.4135, 453.3359	2,3,16-Trihydroxyolean-12-en-28-oic acid + 2Glc + Rha + Xyl
31	18.62	[M + H]^+^	1061.5521	1061.5532	−1.0	C_52_H_84_O_22_	929.5017, 783.4482, 767.4537, 651.4095, 635.4178, 453.3362	2,3,16-Trihydroxyolean-12-en-28-oic acid + Glc + Rha + 2Xyl
32	18.82	[M + Na]^+^	1229.5554	1229.5567	−1.1	C_57_H_90_O_27_	929.4728, 517.3134, 499.3031	Triterpenoid peantaglycoside
33	19.01	[M + Na]^+^	1083.5349	1083.5352	−0.3	C_52_H_84_O_22_	929.5099, 783.4523, 635.4162, 453.3362	2,3,16-Trihydroxyolean-12-en-28-oic acid + Glc + Rha + 2Xyl
34	19.07	[M + Na]^+^	1215.5753	1215.5775	−1.8	C_57_H_92_O_26_	1061.5514, 929.5085, 767.4568, 635.4125, 453.3356	2,3,16-Trihydroxyolean-12-en-28-oic acid + Glc + Rha + 3Xyl
35	19.27	[M + H]^+^	1091.5629	1091.5638	−0.8	C_53_H_86_O_23_	959.5194, 797.4645, 635.4150, 453.3361	2,3,16-Trihydroxyolean-12-en-28-oic acid + 2Glc + Rha + Xyl
36	19.48	[M + Na]^+^	1083.5325	1083.5352	−2.5	C_52_H_84_O_22_	929.5079, 767.4580, 635.4149, 453.3556	2,3,16-Trihydroxyolean-12-en-28-oic acid + Glc + Rha + 2Xyl
37	19.65	[M + H]^+^	1091.5605	1091.5638	−3.0	C_53_H_86_O_23_	797.4366, 503.3375, 485.3260	Triterpenoid tetraglycoside
38	19.79	[M + Na]^+^	833.3936	833.3936	0.0	C_41_H_62_O_16_	679.3704, 517.3165, 499.3063	2,3,16,21-Tetrahydroxyolean-12-en-23,28-dioic acid 28,21-lactone + Glc + Xyl
39	19.96	[M + H]^+^	1061.5521	1061.5532	−1.0	C_52_H_84_O_22_	929.5098, 797.4664, 767.4575, 635.4152, 453.3362	2,3,16-Trihydroxyolean-12-en-28-oic acid + Glc + Rha + 2Xyl
40	20.07	[M + H]^+^	929.5092	929.5110	−1.9	C_47_H_76_O_18_	797.4678, 635.4157, 453.3360	2,3,16-Trihydroxyolean-12-en-28-oic acid + Glc + Rha + Xyl
41	21.08	[M + Na]^+^	981.5063	981.5035	2.9	C_48_H_78_O_19_	819.4191, 635.4167, 453.3372	2,3,16-Trihydroxyolean-12-en-28-oic acid + 2Glc + Rha
42	21.53	[M + H]^+^	1369.6694	1369.6640	3.9	C_64_H_104_O_31_	1237.6249, 1091.5521, 929.5083, 487.3424	2,3,6,16-Tetrahydroxyolean-12-en-28-oic acid + 3Glc + Rha + Xyl
43	21.81	[M + Na]^+^	1377.6309	1377.6303	0.4	C_63_H_102_O_31_	1223.6036, 487.3375	2,3,6,16-Tetrahydroxyolean-12-en-28-oic acid + 3Glc + Rha + Xyl
44	21.89	[M + Na]^+^	967.4510	967.4515	−0.5	C_46_H_72_O_20_	813.4247, 453.3371	Triterpenoid triglycoside
45	22.04	[M + H]^+^	1237.6230	1237.6217	1.1	C_59_H_96_O_27_	1105.5790, 1091.5626, 959.5242, 487.3428	2,3,6,16-Tetrahydroxyolean-12-en-28-oic acid + 3Glc + Rha + Xyl
46	22.14	[M + Na]^+^	951.4934	951.4930	0.4	C_47_H_76_O_18_	635.4149, 453.3368	2,3,16-Trihydroxyolean-12-en-28-oic acid + Glc + Rha + Xyl
47	22.88	[M + Na]^+^	1345.6025	1345.6041	−1.2	C_62_H_98_O_30_	1191.5811, 473.3271	Triterpenoid peantaglycoside
48	22.95	[M + H]^+^	1191.5847	1191.5799	4.0	C_57_H_90_O_26_	1045.5159, 473.3267	Triterpenoid tetraglycoside
49	23.60	[M + Na]^+^	1111.5315	1111.5301	1.3	C_53_H_84_O_23_	957.5078, 473.3265	Triterpenoid tetraglycoside
50	23.78	[M + Na]^+^	1477.6464	1477.6463	0.1	C_67_H_106_O_34_	681.2885, 473.3246	Triterpenoid hexaglycoside
51	24.08	[M + H]^+^	1455.6688	1455.6644	3.0	C_67_H_106_O_34_	1323.6157, 473.3232	Triterpenoid hexaglycoside
52	24.13	[M + H]^+^	1365.6348	1365.6327	1.5	C_64_H_100_O_31_	1191.5837, 1045.5164, 487.3355	Triterpenoid hexaglycoside
53	24.22	[M + H]^+^	1177.5974	1177.6006	−2.7	C_57_H_92_O_25_	1045.5479, 487.3419	Triterpenoid peantaglycoside
54	24.35	[M + Na]^+^	1081.5162	1081.5162	−3.1	C_52_H_82_O_22_	751.3569, 487.3407	2,3,16-Trihydroxyolean-11,13-dien-28-oic acid + Glc + Rha + 2Xyl
55	25.28	[M + H]^+^	1365.6355	1365.6327	2.1	C_64_H_100_O_31_	1191.5891, 473.3268	Triterpenoid peantaglycoside
56	25.47	[M + H]^+^	1455.7042	1455.7008	2.3	C_68_H_110_O_33_	1191.6173, 487.3420	Triterpenoid hexaglycoside
57	25.86	[M + H]^+^	1265.6177	1265.6166	0.9	C_60_H_96_O_28_	1133.5748, 987.5201, 487.3414	2,3,6,16-Tetrahydroxyolean-12-en-28-oic acid + 3Glc + Rha + Xyl
58	25.98	[M + H]^+^	1323.6268	1323.6221	3.6	C_62_H_98_O_30_	1191.5845, 1059.5306, 487.3489	2,3,16-Trihydroxyolean-11,13-dien-28-oic acid + Glc + Rha + 4Xyl
59	26.26	[M + H]^+^	1411.6802	1411.6745	4.0	C_66_H_106_O_32_	1279.6354, 487.3660	2,3,6,16-Tetrahydroxyolean-12-en-28-oic acid + 3Glc + Rha + Xyl
60	26.37	[M + Na]^+^	1345.6036	1345.6041	−0.4	C_62_H_98_O_30_	1191.5786, 1059.5397, 487.3437	2,3,16-Trihydroxyolean-11,13-dien-28-oic acid + Glc + Rha + 4Xyl
61	26.55	[M + Na]^+^	1419.6461	1419.6408	3.7	C_65_H_104_O_32_	1265.6193. 487.3432	2,3,6,16-Tetrahydroxyolean-12-en-28-oic acid + 3Glc + Rha + Xyl
62	26.80	[M + Na]^+^	1301.5820	1301.5778	3.2	C_60_H_94_O_29_	1147.5524, 985.5131, 487.3415	Triterpenoid peantaglycoside
63	28.88	[M + Na]^+^	1431.6439	1431.6408	2.2	C_66_H_104_O_32_	1277.6174, 1131.5646, 983.5145, 453.3371	Triterpenoid hexaglycoside
64	29.90	[M + H]^+^	1453.6885	1453.6851	2.3	C_68_H_108_O_33_	1321.6450, 487.3407	Triterpenoid hexaglycoside
65	30.19	[M + H]^+^	1439.6742	1439.6695	3.3	C_67_H_106_O_33_	1307.6295, 487.3424	2,3,6,16-Tetrahydroxyolean-12-en-28-oic acid + 3Glc+ Rha + Xyl
66	30.41	[M + H]^+^	1453.6913	1453.6821	4.3	C_68_H_108_O_33_	1321.6445, 487.3423	Triterpenoid hexaglycoside
67	30.71	[M + H]^+^	1439.6765	1439.6695	4.9	C_67_H_106_O_33_	1307.6289, 487.3435	2,3,6,16-Tetrahydroxyolean-12-en-28-oic acid + 3Glc + Rha + Xyl
68	32.45	[M + H]^+^	1305.6506	1305.6479	2.1	C_63_H_100_O_28_	1173.6053, 1027.5429, 487.3442	Triterpenoid peantaglycoside

Abbreviations: Glc, glucose; Rha, rhamnose; Xyl, xylose.

## Data Availability

The data supporting the conclusions of this article are included within the article. Raw data are available from the corresponding author upon reasonable request.

## References

[1] Chandler M., Cunningham S., Lund E.M., Khanna C., Naramore R., Patel A., Day M.J. (2017). Obesity and Associated Comorbidities in People and Companion Animals: A One Health Perspective. J. Comp. Pathol..

[2] German A.J. (2006). The growing problem of obesity in dogs and cats. J. Nutr..

[3] Gilor C., Graves T.K. (2023). Diabetes Mellitus in Cats and Dogs. Vet. Clin. N. Am.-Small Anim. Pract..

[4] Misha M.S., Destrumelle S., Le Jan D., Mansour N.M., Fizanne L., Ouguerram K., Desfontis J.C., Mallem M.Y. (2024). Preventive effects of a nutraceutical mixture of berberine, citrus and apple extracts on metabolic disturbances in Zucker fatty rats. PLoS ONE.

[5] Wu H., Ballantyne C.M. (2020). Metabolic Inflammation and Insulin Resistance in Obesity. Circ. Res..

[6] Webb T.L., Molina J., Sheridan L., du Plessis H., Brown J., Abraham H., Morton O., Mckay S. (2025). Developing and evaluating a health pack to support dog owners to manage the weight of their companion animals. Front. Vet. Sci..

[7] Larsen J.A., Villaverde C. (2016). Scope of the Problem and Perception by Owners and Veterinarians. Vet. Clin. N. Am.-Small Anim. Pract..

[8] Alkhatib A., Tsang C., Tiss A., Bahorun T., Arefanian H., Barake R., Khadir A., Tuomilehto J. (2017). Functional Foods and Lifestyle Approaches for Diabetes Prevention and Management. Nutrients.

[9] van Amersfort K., van der Lee A., Hagen-Plantinga E. (2023). Evidence-base for the beneficial effect of nutraceuticals in canine dermatological immune-mediated inflammatory diseases—A literature review. Vet. Dermatol..

[10] Su X.D., Jang H.J., Wang C.Y., Lee S.W., Rho M.C., Kim Y.H., Yang S.Y. (2019). Anti-inflammatory Potential of Saponins from via NF-κB/MAPK Activation. J. Nat. Prod..

[11] Du F., Huang R., Lin D., Wang Y., Yang X., Huang X., Zheng B., Chen Z., Huang Y., Wang X. (2021). Resveratrol Improves Liver Steatosis and Insulin Resistance in Non-alcoholic Fatty Liver Disease in Association with the Gut Microbiota. Front. Microbiol..

[12] Barazzoni R., Bischoff S.C., Busetto L., Cederholm T., Chourdakis M., Cuerda C., Delzenne N., Genton L., Schneider S., Singer P. (2022). Nutritional management of individuals with obesity and COVID-19: ESPEN expert statements and practical guidance. Clin. Nutr..

[13] He L., Su Z., Wang S. (2024). The anti-obesity effects of polyphenols: A comprehensive review of molecular mechanisms and signal pathways in regulating adipocytes. Front. Nutr..

[14] Sayed U.F.S.M., Moshawih S., Goh H.P., Kifli N., Gupta G., Singh S.K., Chellappan D.K., Dua K., Hermansyah A., Ser H.L. (2023). Natural products as novel anti-obesity agents: Insights into mechanisms of action and potential for therapeutic management. Front. Pharmacol..

[15] Nagao T., Okabe H., Yamauchi T. (1988). Studies on the Constituents of *Aster tataricus* L. f. I.: Structures of Shionosides a and B: Monoterpene Glycosides Isolated from the Root. Chem. Pharm. Bull..

[16] Nagao T., Okabe H., Yamauchi T. (1990). Studies on the Constituents of *Aster tataricus* L. f. III.: Structures of Aster Saponins E and F Isolated from the Root. Chem. Pharm. Bull..

[17] Dongliang C., Yu S. (1993). Terpenoid glycosides from the roots of Aster tataricus. Phytochemistry.

[18] Hoenig M. (2014). Comparative Aspects of Human, Canine, and Feline Obesity and Factors Predicting Progression to Diabetes. Vet. Sci..

[19] Guo X., Wang Y., Zhu Z., Li L. (2024). The Role of Plant Extracts in Enhancing Nutrition and Health for Dogs and Cats: Safety, Benefits, and Applications. Vet. Sci..

[20] Roudebush P., Schoenherr W.D., Delaney S.J. (2008). Timely topics in nutrition—An evidence-based review of the use of nutraceuticals and dietary supplementation for the management of obese and overweight pets. Javma-J. Am. Vet. Med. A.

[21] Lee H.S., Jung J.I., Hwang J.S., Hwang M.O., Kim E.J. (2023). Cydonia oblonga Miller fruit extract exerts an anti-obesity effect in 3T3-L1 adipocytes by activating the AMPK signaling pathway. Nutr. Res. Pract..

[22] Han S., Oh K.S., Yoon Y., Park J.S., Park Y.S., Han J.H., Jeong A.L., Lee S., Park M., Choi Y.A. (2011). Herbal extract THI improves metabolic abnormality in mice fed a high-fat diet. Nutr. Res. Pract..

[23] Nakamura A., Terauchi Y. (2013). Lessons from Mouse Models of High-Fat Diet-Induced NAFLD. Int. J. Mol. Sci..

[24] Recena Aydos L., Aparecida do Amaral L., Serafim de Souza R., Jacobowski A.C., Freitas Dos Santos E., Rodrigues Macedo M.L. (2019). Nonalcoholic Fatty Liver Disease Induced by High-Fat Diet in C57bl/6 Models. Nutrients.

[25] Yoon A., Tammen S.A., Park S., Han S.N., Choi S.W. (2017). Genome-wide hepatic DNA methylation changes in high-fat diet-induced obese mice. Nutr. Res. Pract..

[26] Wang C.Y., Liao J.K. (2012). A mouse model of diet-induced obesity and insulin resistance. Methods Mol. Biol..

[27] Lin Y.G., Ren N.N., Li S.Y., Chen M., Pu P. (2019). Novel anti-obesity effect of scutellarein and potential underlying mechanism of actions. Biomed. Pharmacother..

[28] Drummer C., Saaoud F., Jhala N.C., Cueto R., Sun Y., Xu K.M., Shao Y., Lu Y.F., Shen H.M., Yang L. (2023). Caspase-11 promotes high-fat diet-induced NAFLD by increasing glycolysis, OXPHOS, and pyroptosis in macrophages. Front. Immunol..

[29] Matthews D.R., Hosker J.P., Rudenski A.S., Naylor B.A., Treacher D.F., Turner R.C. (1985). Homeostasis model assessment: Insulin resistance and beta-cell function from fasting plasma glucose and insulin concentrations in man. Diabetologia.

[30] Baek Y.B., Kwon H.J., Sharif M., Lim J., Lee I.C., Ryu Y.B., Lee J.I., Kim J.S., Lee Y.S., Kim D.H. (2022). Therapeutic strategy targeting host lipolysis limits infection by SARS-CoV-2 and influenza A virus. Signal Transduct. Target. Ther..

[31] Takeuchi A., Takeuchi M., Oikawa K., Sonoda K.H., Usui Y., Okunuki Y., Takeda A., Oshima Y., Yoshida K., Usui M. (2009). Effects of dioxin on vascular endothelial growth factor (VEGF) production in the retina associated with choroidal neovascularization. Investig. Ophthalmol. Vis. Sci..

[32] Kleiner D.E., Brunt E.M., Van Natta M., Behling C., Contos M.J., Cummings O.W., Ferrell L.D., Liu Y.C., Torbenson M.S., Unalp-Arida A. (2005). Design and validation of a histological scoring system for nonalcoholic fatty liver disease. Hepatology.

[33] Mirabelli M., Misiti R., Sicilia L., Brunetti F.S., Chiefari E., Brunetti A., Foti D.P. (2024). Hypoxia in Human Obesity: New Insights from Inflammation towards Insulin Resistance—A Narrative Review. Int. J. Mol. Sci..

[34] Kang G.-S., Jo H.-J., Lee Y.-R., Oh T., Park H.-J., Ahn G.O. (2023). Sensing the oxygen and temperature in the adipose tissues—Who’s sensing what?. Exp. Mol. Med..

[35] Moon Y.A. (2017). The SCAP/SREBP Pathway: A Mediator of Hepatic Steatosis. Endocrinol. Metab..

[36] Ferré P., Foufelle F. (2010). Hepatic steatosis: A role for lipogenesis and the transcription factor SREBP-1c. Diabetes Obes. Metab..

[37] Millán J., Pintó X., Muñoz A., Zúñiga M., Rubiés-Prat J., Pallardo L.F., Masana L., Mangas A., Hernández-Mijares A., González-Santos P. (2009). Lipoprotein ratios: Physiological significance and clinical usefulness in cardiovascular prevention. Vasc. Health Risk Man..

[38] Rains T.M., Agarwal S., Maki K.C. (2011). Antiobesity effects of green tea catechins: A mechanistic review. J. Nutr. Biochem..

[39] Aloo S.O., Ofosu F.K., Kim N.H., Kilonzi S.M., Oh D.H. (2023). Insights on Dietary Polyphenols as Agents against Metabolic Disorders: Obesity as a Target Disease. Antioxidants.

[40] German A.J. (2016). Weight management in obese pets: The tailoring concept and how it can improve results. Acta Vet. Scand..

[41] Porsani M.Y.H., Teixeira F.A., Amaral A.R., Pedrinelli V., Vasques V., de Oliveira A.G., Vendramini T.H.A., Brunetto M.A. (2020). Factors associated with failure of dog’s weight loss programmes. Vet. Med. Sci..

[42] Broome H.A.O., Woods-Lee G.R.T., Flanagan J., Biourge V., German A.J. (2023). Weight loss outcomes are generally worse for dogs and cats with class II obesity, defined as >40% overweight. Sci. Rep..

[43] Jang J.H., Sung J.H., Huh J.Y. (2025). Diverse Functions of Macrophages in Obesity and Metabolic Dysfunction-Associated Steatotic Liver Disease: Bridging Inflammation and Metabolism. Immune Netw..

[44] Quang T.H., Nguyen T.T.N., Minh C.V., Kiem P.V., Thao N.P., Tai B.H., Nhiem N.X., Song S.B., Kim Y.H. (2011). Effect of triterpenes and triterpene saponins from the stem bark of on the transactivational activities of three PPAR subtypes. Carbohyd. Res..

[45] Li W., Yan X.T., Sun Y.N., Ngan T.T., Shim S.H., Kim Y.H. (2014). Anti-Inflammatory and PPAR Transactivational Effects of Oleanane-Type Triterpenoid Saponins from the Roots of Pulsatilla koreana. Biomol. Ther..

[46] Yao P.Y., Liu Y.J. (2023). Terpenoids: Natural Compounds for Non-Alcoholic Fatty Liver Disease (NAFLD) Therapy. Molecules.

[47] Qiu L., Feng R.B., Wu Q.S., Wan J.B., Zhang Q.W. (2023). Total saponins from Panax japonicus attenuate acute alcoholic liver oxidative stress and hepatosteatosis by p62-related Nrf2 pathway and AMPK-ACC/PPARα axis in vivo and in vitro. J. Ethnopharmacol..

[48] Peng F., Ren X., Du B., Yang Y. (2023). Pyrus ussuriensis Maxim 70% ethanol eluted fraction ameliorates inflammation and oxidative stress in LPS-induced inflammation in vitro and in vivo. Food Sci. Nutr..

[49] Bi Y.M., Wu Y.T., Chen L., Tan Z.B., Fan H.J., Xie L.P., Zhang W.T., Chen H.M., Li J., Liu B. (2018). 3,5-Dicaffeoylquinic acid protects H9C2 cells against oxidative stress-induced apoptosis via activation of the PI3K/Akt signaling pathway. Food Nutr. Res..

[50] Oh J.H., Karadeniz F., Lee J.I., Seo Y., Kong C.S. (2019). Protective effect of 3,5-dicaffeoyl-epi-quinic acid against UVB-induced photoaging in human HaCaT keratinocytes. Mol. Med. Rep..

[51] Ding X., Jian T., Li J., Lv H., Tong B., Li J., Meng X., Ren B., Chen J. (2020). Chicoric Acid Ameliorates Nonalcoholic Fatty Liver Disease via the AMPK/Nrf2/NFkappaB Signaling Pathway and Restores Gut Microbiota in High-Fat-Diet-Fed Mice. Oxid. Med. Cell. Longev..

[52] Wang L., Duan J., Jia N., Liu M., Cao S., Weng Y., Zhang W., Cao J., Li R., Cui J. (2021). IRS-2/Akt/GSK-3beta/Nrf2 Pathway Contributes to the Protective Effects of Chikusetsu Saponin IVa against Lipotoxicity. Oxid. Med. Cell. Longev..

[53] Xu Y., Bai L., Yang X., Huang J., Wang J., Wu X., Shi J. (2024). Recent advances in anti-inflammation via AMPK activation. Heliyon.

[54] Kubota N., Kubota T., Kajiwara E., Iwamura T., Kumagai H., Watanabe T., Inoue M., Takamoto I., Sasako T., Kumagai K. (2016). Differential hepatic distribution of insulin receptor substrates causes selective insulin resistance in diabetes and obesity. Nat. Commun..

[55] Cavaliere G., Cimmino F., Trinchese G., Catapano A., Petrella L., D’Angelo M., Lucchin L., Mollica M.P. (2023). From Obesity-Induced Low-Grade Inflammation to Lipotoxicity and Mitochondrial Dysfunction: Altered Multi-Crosstalk between Adipose Tissue and Metabolically Active Organs. Antioxidants.

[56] del Hierro J.N., Herrera T., Fornari T., Reglero G., Martin D. (2018). The gastrointestinal behavior of saponins and its significance for their bioavailability and bioactivities. J. Funct. Foods.

[57] Luo Z.C., Xu W.C., Zhang Y., Di L.Q., Shan J.J. (2020). A review of saponin intervention in metabolic syndrome suggests further study on intestinal microbiota. Pharmacol. Res..

[58] Teng Z.H., Yuan C.J., Zhang F., Huan M.L., Cao W.D., Li K.C., Yang J.Y., Cao D.Y., Zhou S.Y., Mei Q.B. (2012). Intestinal Absorption and First-Pass Metabolism of Polyphenol Compounds in Rat and Their Transport Dynamics in Caco-2 Cells. PLoS ONE.

[59] Yang X.W., Wang N., Li W., Xu W., Wu S. (2013). Biotransformation of 4,5--dicaffeoylquinic acid methyl ester by human intestinal flora and evaluation on their inhibition of NO production and antioxidant activity of the products. Food Chem. Toxicol..

[60] Shin J.-W., Seol I.-C., Son C.-G. (2010). Interpretation of Animal Dose and Human Equivalent Dose for Drug Development. J. Korean Orient. Med..

[61] Nair A.B., Jacob S. (2016). A simple practice guide for dose conversion between animals and human. J. Basic Clin. Pharm..

[62] Nishikawa S., Yasoshima A., Doi K., Nakayama H., Uetsuka K. (2007). Involvement of sex, strain and age factors in high fat diet-induced obesity in C57BL/6J and BALB/cA mice. Exp. Anim..

[63] Van Herck M.A., Vonghia L., Francque S.M. (2017). Animal Models of Nonalcoholic Fatty Liver Disease—A Starter’s Guide. Nutrients.

[64] Gomes Schmitt E., Erminda Schreiner G., Smolski dos Santos L., Pereira de Oliveira C., Berny Pereira C., Muller de Moura Sarmento S., Klock C., Casanova Petry C., Denardin E.L.G., Gonçalves I.L. (2025). Moro Orange (*Citrus sinensis* (L.) Osbeck) Extract Mitigates Metabolic Dysregulation, Inflammation, Oxidative Stress, and Adipose Tissue Hyperplasia in Obese Rats. Int. J. Mol. Sci..

[65] Jolliffe I.T., Cadima J. (2016). Principal component analysis: A review and recent developments. Philos. Trans. R. Soc. A.

[66] Lee Y.H., Kim Y.-S., Song M., Lee M., Park J., Kim H. (2015). A Herbal Formula HT048, Citrus unshiu and Crataegus pinnatifida, Prevents Obesity by Inhibiting Adipogenesis and Lipogenesis in 3T3-L1 Preadipocytes and HFD-Induced Obese Rats. Molecules.

